# Wasp pollination and pollinator filtering by dense hairs at the floral tube entrance in *Marsdenia tinctoria* (Apocynaceae)

**DOI:** 10.1007/s10265-025-01621-z

**Published:** 2025-02-20

**Authors:** Ko Mochizuki, Ayako Watanabe-Taneda

**Affiliations:** 1 Botanical Gardens, Graduate School of Science, The University of Tokyo, Tokyo, 112-0001 Japan; 2 Department of Biological Sciences, Graduate School of Science, The University of Tokyo, 7-3-1, Hongo, Bunkyo, Tokyo, 113-0033 Japan

**Keywords:** Asclepiadoideae, Floral filter, Floral hair, Flower color change, Pollination, Wasp

## Abstract

The physical filtering of pollinators is an important factor influencing pollination effectiveness. This study explored the potential functions of dense hairs that completely obstruct the entrance of floral tube in *Marsdenia tinctoria* by characterizing the flowers of this species, as well as its pollinators and their behavior. The corolla was white upon blooming in the morning, then turned yellow at night, and the flower finally dropped by the third morning. The hairs tended to be disheveled in yellow-petaled flowers. Pollination success increased with floral age. Direct observations of flowers in natural *M*. *tinctoria* populations over a period of 32 h recorded 126 visitors, of which 70% were wasps. We observed pollinia attached to the mouthparts of wasps, carpenter bees, and honeybees, but not to those of butterflies, moths, flies, or ants. Detailed examination of insect mouthparts and floral morphology indicated that insect visitors that acted as pollinators had mouthparts longer than the floral tubes, equipped with hairs to which pollinia could attach. The mouthparts of potter wasps were often covered with pollinaria, carrying on average 30–75 pollinia. The dense floral hairs were penetrated by large-bodied visitors, and blocked smaller visitors. Disturbance of these floral hairs allowed smaller insects to access nectar, suggesting that the hairs function in preventing nectar theft by smaller insects. This study presents the first case of wasp pollination in the genus *Marsdenia* and provides insights into the potential function of its dense floral hairs, a synapomorphy of this genus, in filtering floral visitors.

**Supplementary Information:**

The online version contains supplementary material available at 10.1007/s10265-025-01621-z.

## Introduction

Effective pollen transfer is a key factor determining reproductive success in flowering plants. The number of animal species involved in plant pollination varies from 1 to over 100, depending on the plant species, producing the spectrum from specialist to generalist pollination systems (Olesen et al. 2007; Willmer 2011). Not all floral visitors contribute equally to pollination success due to their variable mechanical and behavioral fit with the flowers (Mayfield et al. 2001). Therefore, eliminating less important or unnecessary floral visitors that waste nectar and pollen likely contributes to preventing a reduction in fitness (Muchhala et al. 2010; Takeda et al. 2021). Limiting access to certain pollinators is achieved through both pre- and post-attraction processes. Plants often selectively attract pollinators using specific cues such as floral display color and scent (Raguso 2008; Raven 1972). In addition, secondary metabolites in their nectar can deter visitors through learned avoidance (Johnson et al. 2006; Martos et al. 2015; Shuttleworth and Johnson 2009).

Some floral visitors are unable to contribute to pollination as they are filtered out by floral morphology and orientation through physiological and behavioral mechanisms (Armbruster 2017). Long floral tubes are adapted to allow access to long-proboscid pollinators, while deterring short-proboscid pollinators (Johnson et al. 2017). The size, morphology, and spatial arrangement of tepals, stigmas, and anthers impose limits on pollinator size (Martos et al. 2015; Mochizuki et al. 2017; Shuttleworth et al. 2017). Complex floral morphology allows a handful of insects such as bumblebees to access floral resources (Laverty and Plowright 1988). Similarly, downward-oriented flowers can deter visitation by certain taxa, such as hoverflies (Wang et al. 2014). The slippery outer corolla surfaces of the campanulate flowers of *Codonopsis lanceolata* and *Fritillaria koidzumiana* filter out ants that discourage visitation by desired pollinators (Takeda et al. 2021). These floral traits have evolved independently among angiosperms, suggesting the importance of deterring ineffective floral visitors (Armbruster 2017).

Despite the important role of floral architecture in filtering pollinators, the functions of smaller tissues within the flower remain unclear. Bumblebees are reported to prefer flowers with conical epidermal cells, which improve their grip (Papiorek et al. 2014; Whitney et al. 2009). Epidermal cells can be arranged into hairs called trichomes. Trichomes are commonly found on flowers, where they can encourage insect pollination by secreting nectar (Johnson et al. 2007), presenting an appealing display or a guiding structure (Dufy and Johnson 2015; Kunze 1991), mimicking stamens (to encourage hoverflies to linger: Tagawa 2023), or trapping insects (e.g. tube-flowered *Aristolochia* and *Ceropegia* species: Oelschlägel et al. 2009; Ollerton et al. 2017). Although several studies have suggested that these hairs may protect the flower from nectar-thieving ants, very few studies have examined this function (Tagawa 2018). Therefore, the role of floral hairs as a mechanism for filtering pollinators remains relatively unexplored, despite its frequent observation across plant taxa.

**Fig. 1:**
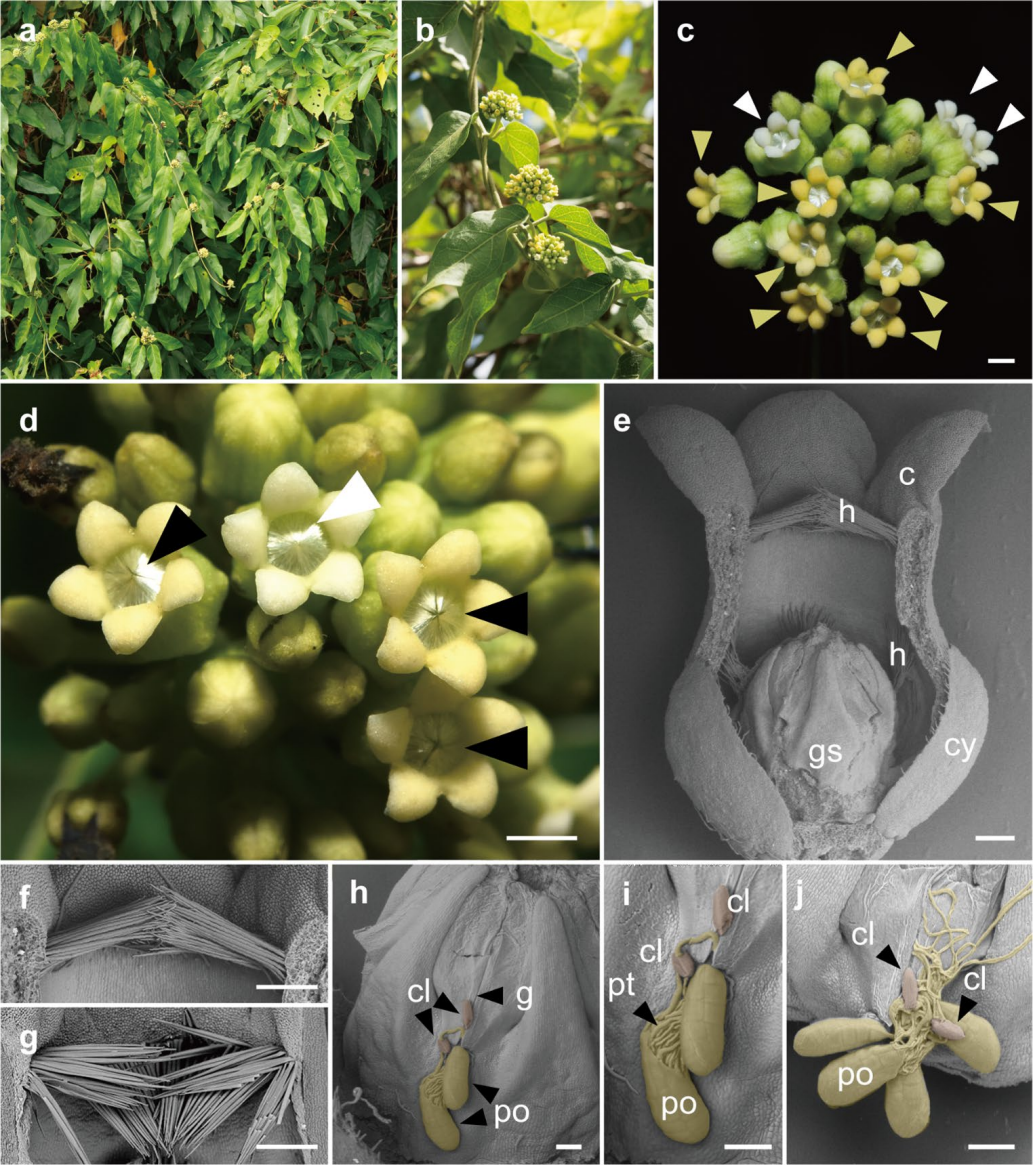
Images of *Marsdenia tinctoria*. (**a**) Flowering individual hanging over vegetation (*Mallotus philippensis*). (**b**) Inflorescences. (**c**) Close-up view of an inflorescence containing white- and yellow-petaled flowers, indicated by arrowheads. (**d**) Close-up view of flowers. Dense hairs obstruct the entrance of the floral tube; these hairs are aligned in the white flower (white triangles) but disturbed in yellowish flowers (black triangles). (**e**) Internal structure of a flower, with the front corolla removed. (**f**) and (**g**) Intact and disturbed hairs at the entrance of the floral tube. (**h**) Gynostegium with pollinia received. (**i**) Close-up view of deposited pollinia, from which pollen tubes germinated. (**j**) Gynostegium receiving multiple pollinia. Abbreviations: c, corolla; cl, corpusculum (clip); cy, calyx; g, guide rail; gs, gynostegium; h, hair; po, pollinium; pt, pollen tube. Scale bars: 1 mm for (**c**) and (**d**), 0.25 mm for (**e**)–(**g**), 0.1 mm for (**h**)–(**j**)

*Marsdenia tinctoria* R.Br. (Asclepiadoideae, Apocynaceae) is a perennial twining plant distributed in the Himalayas and Southeast Asian countries, whose northernmost range extends to the Ryukyu Islands of Japan (Fig. 1a, b). This plant is ethnobotanically important for its indigo-like chemicals, which are used as a dye throughout its distribution range (Teron and Borthakur 2012). The flowers of *M*. *tinctoria* possess a tubular corolla (depth, 2–3 mm), which is colored white or yellow (Fig. 1c). The entrance of its corolla tube is obstructed by dense, needle-like solid hairs (Fig. 1d), such that the interior of the flower cannot be seen from outside. These hairs are difficult to extract from the corolla using tweezers, and quickly return to their original shape and position after manipulation, demonstrating their role as a strong barrier. Although the presence of these dense hairs on the petals is a defining characteristic of genus *Marsdenia* (Liede-Schumann et al. 2022), their function is unknown.

This study explored the potential function of these dense hairs by characterizing the flowers of *M*. *tinctoria*, as well as its insect pollinators and their behaviors. Our observations provide insights into the role of dense corolla hairs in filtering floral visitors that may not contribute to pollination in *M*. *tinctoria*.

## Materials and methods

### Floral traits of Asclepiadoideae species

Plants within the subfamily Asclepiadoideae (Apocynaceae) have elaborate floral traits; for example, the stamen and pistil are transformed and fused to form a gynostegium and pollen grains clump to form pollinia (Endress et al. 2018). In these species, each flower produces five pollinaria, each composed of two pollinia connected to a clip-like corpusculum via translator arms known as caudicles. The gynostegium is a structure that contains the five pollinaria, forming five stigmatic chambers from which many species secrete nectar (Monteiro and Demarco 2017). Each stigmatic chamber is concealed within a slit bounded by two “guide rails”. When a pollinator moves a body part through the slit into the pollinarium located just above it, the corpusculum attaches to the insect body, stripping the pollinia away from the slit; through repetition, this process results in pollen deposition on the stigma (Wyatt and Broyles 1994). Normally, only one pollinium is deposited onto the stigma at each sequence, while the corpusculum and the second pollinium remain attached to the insect body.

Owing to this mechanical process, only visitors morphologically and behaviorally compatible with the traits of a given flower can successfully detach and carry pollinia (Mochizuki et al. 2017; Ollerton and Liede-Schumann 1997). Therefore, if pollinia are found attached to the body of an insect, it is considered to have the potential to contribute to pollination.

### Floral features

In preliminary experiments, we observed yellow and white corollas (Fig. 1b, c), which appeared to represent a floral color change associated with the age and/or pollination state of the flower (Ohashi et al. 2015; Weiss 1995). Because such floral color changes have not been reported in the genus *Marsdenia*, we investigated whether pollinator visitation may have triggered a shift from white to yellow. We covered the inflorescences of four individuals with meshed bags (1 mm mesh size) at 18:00 on July 28, 2016 to exclude floral visitors and observe changes in flower color. Initially, a total of 30 white flowers and 10 yellow flowers were observed; most flowers on the inflorescences were in the budding stage. The flowers were monitored at 10:00, 18:00, and 23:00 on July 29, 2016.

The inner morphology of the flowers, including hairs at the entrance of the floral tube and the pollinated gynostegia, was observed by scanning electron microscopy (SEM) using TM3000 (Hitachi, Tokyo, Japan) and JSM-6510LV (JEOL Ltd. Tokyo, Japan) microscopes. Flowers were collected in the field and kept in 70% ethanol. The flower samples were soaked in a series of diluted ethanol solutions for ethanol removal, and then cryo-fixed in liquid nitrogen prior to SEM observation using a cryo-sample holder (SA1202, JEOL Ltd.). Observations were conducted under low-vacuum conditions of 30 Pa, 15.0 kV, and accelerating voltage.

Because floral visitors exhibited nectar-feeding behavior, we measured nectar volume. Inflorescences consisting solely of buds were covered with mesh bags (1 mm mesh size) at 18:00 on July 28, 2016. Preliminary observations suggested that the flowers opened in the morning; therefore, inflorescences were checked the morning after bagging to confirm flowering. The bags remained in place until 18:00 on July 29, 2016. Nectar was extracted from flowers that had opened by morning using 1 µL glass capillary tubes. In total, nine flowers were sampled from two individuals.

### Observation of floral visitors and their behavior

Pollinators were observed in plant populations near the Itokazu Castle ruins in Okinawa Prefecture on 28 July 2014, 26–29 July 2016, and 16–19 August 2018 (Table S1). Observations were conducted from 09:30 to 20:30, covering the time range when diurnal and nocturnal insects are active. The behavior of floral visitors was observed and the insects were captured whenever possible. Some floral visitors were photographed using a Nikon D7200 camera (Nikon, Tokyo, Japan) equipped with a NIKKOR 60 mm micro lens (AF-S; f/2.8G ED; Nikon) to examine their behavior in detail. The behavior of moths, dipterans, and ants (ineffective as pollinators: Table 1) was carefully observed whenever possible to determine which colored flower they visited and whether the visitation was successful. Visitation attempts to white flowers were counted as the number of events rather than the number of individuals.

**Table 1:** Body size and pollinarium attachment in floral visitors to *Marsdenia tinctoria*

Order	Family	Species	Number of individuals			Number of corpuscula attached (average ± SD)
			Observed	Inspected	Carrying corpuscula	
Hymenoptera						
	Vespidae (Eumeninae)					
		*Anterhynchium flavomarginatum*	46	40	40	29.5 ± 17.9
		*Rhynchium quinquecinctum*	5	5	5	32.8 ± 20.5
	Vespidae (Polistinae)					
		*Polistes jokahamae*	30	25	15	1.83 ± 2.17
	Scoliidae					
		*Scolia melanosoma*	1	1	1	2
		Scoliidae sp.	1	0		
	Apidae					
		*Apis mellifera*	1	1	1	9
		*Xylocopa flavifrons*	7	5	4	9.8 ± 6.65
	Formicidae					
		*Camponotus bishamon*	3	0		
		*Technomyrmex brunneus*	7	0		
Lepidoptera						
	Brachodidae					
		*Nigilgia limata*	10	10	0	
	Papilionidae					
		*Graphium sarpedon*	5	4	0	
	Pyraloidea					
		*Pyraloidea* sp.	2	2	0	
	Sphingidae					
		*Macroglossum* sp.	2	0		
Diptera						
	Ceraropogonidae					
		*Formicinae* sp.	2	0		
	Syrphidae					
		*Eristalinus quinquestriatus*	2	2	0	
	Calliphoridae					
		*Stomorhina obsoleta*	2	1	0	

SD, standard deviation

Furthermore, we selected three white flowers with undisturbed hairs and manually disrupted the hairs by repeatedly bending them with tweezers. Then one individual of *Nigilgia limata* was gently introduced to the inflorescence using an aspirator, letting it visit the manipulated flowers. The behavior of the *N. limata* individual to these manipulated flowers was then observed.

### Evaluation of the importance of floral visitors as pollinators

Because floral visitors may not contribute equally to pollination, it is necessary to evaluate their contributions by taxonomic group to understand their pollination ecology. We quantified pollinia attached to the body of each floral visitor, and recorded their specific locations on the body through stereomicroscopy. Because many pollinia could attach to the proboscis, clouding the image, we were unable to conduct accurate pollinium counts. As the corpuscula are normally not deposited on the flowers in Asclepiadoideae, we counted these as a proxy of pollinator effectiveness (But some of the corpuscula attached to the pollinators seemed lost in *M*. *tinctoria*: see Results). To gain high-quality images of pollinia attachment, close-up photographs of the heads and glossae of floral visitors were obtained through focus stacking. A Nikon Z8 camera (Nikon Corporation, Tokyo, Japan) was used, fitted with either a NIKKOR Z MC 105 mm f/2.8 VR S lens (Nikon Corporation, Tokyo, Japan) or a NIKKOR Z 24–120 mm f/4 S lens (Nikon Corporation, Tokyo, Japan), with an Olympus PLN4 objective lens (Evident Corporation, Tokyo, Japan) mounted at the front. The Z8’s built-in focus shift function was used to capture a series of 70 to 100 consecutive images, which were subsequently focus-stacked using the depth map method in Helicon Focus 8.2.17 (Helicon Soft, Kharkiv, Ukraine). Further, the detailed placement of pollinia attached to the insect body was studied using SEM (JSM-6510LV; JEOL, Ltd.).

To investigate the relationship between floral tube depth and pollen transport effectiveness by pollinators, we measured flower tube depth and pollinator mouthpart length in hymenopteran insects carrying pollinia. Flowers were collected from four plants, and five flowers from each plant were preserved in ethanol until measurement. The floral tube depth was defined as the distance from the floral hairs to the base of the corolla. Observations of flower-visiting behavior suggested that the insects inserted their mandibles into the flower base to drink the accumulated nectar. Therefore, the length from the mandible to the tip of the extended glossa was measured as a representation of the functional mouthparts. The samples were photographed under a stereomicroscope, and measurements on the resulting images were conducted using ImageJ v1.48 (Schneider et al. 2012).

Analysis of variance (ANOVA) was performed to compare measurements among pollinator species, and a linear model was used to determine whether differences in pollinium numbers could be explained by pollinator species, mouthpart length, or their interaction. Akaike information criterion (AIC) values of the three models were compared, and the model with the lowest AIC value was further analyzed using a linear model. For the most frequent floral visitor, *Anterhynchium flavomarginatum*, the relationship between mouthpart length and pollinium number was also investigated using a linear model. All analyses were performed using the “lm” function in the *MASS* (Venables and Ripley 2002) package in R v3.3.3 (R Core Team 2017).

### Pollen transfer efficiency and floral color

The frequencies of pollinarium removal and pollinium insertion into the stigmatic slit were determined. Flowers were collected at 18:00 on 29 July 2016 and preserved in ethanol for later microscopic inspection. Using three flowering individuals, sampling was conducted randomly on flowers regardless of flower colors. Mean pollinarium removal and pollinium insertion rates were calculated by pooling data from these three individuals. Pollen transfer efficiency (PTE) was calculated as the proportion of removed pollinia that were subsequently deposited to the stigmas, i.e., the total number of inserted pollinia divided by the total number of removed pollinia, based on the assumption of two pollinia per pollinarium (Shuttleworth and Johnson 2009).

During preliminary observations, flower color was assumed to change with age. Pollen transfer efficiency was investigated separately for both white and yellow flowers. Flowers were randomly collected from four individuals, and 30 flowers of each color were inspected. Differences in the number of pollinia removed and inserted were compared between flower colors using the Mann–Whitney U test.

## Results

### Floral features

Inflorescences generally contained both white and yellow flowers, which can be clearly distinguished (Fig. 1c). By 10:00 on the morning after flowering, the corollas of all but 1 of the 30 flowers initially recorded as white had turned yellow, and all 10 flowers initially recorded as yellow had dropped. In addition, 34 new flowers had bloomed, all of which remained white at 18:00 and had begun to turn yellowish by 23:00. Therefore, it seemed that flowers started to turn yellowish in the evening, and their yellow color became more pronounced by the morning.

SEM observations revealed numerous needle-like hairs growing towards the center of the entrance of the floral tube (Fig. 1e). In white flowers, these hairs grew slightly upward and obliquely toward the center of the flower (Fig. 1f). However, in yellow flowers, which appeared to have been visited by pollinators, the hairs were bent from the base and pointed downward (Fig. 1g). In addition, hairs were observed within the floral tube around the gynostegium (Fig. 1e).

In pollinated flowers, pollinia were trapped by the gynostegium. In some flowers, the entire pollinarium was inserted, without separation of the pollinia, or multiple pollinaria were inserted (Fig. 1i, j). These pollinaria were not deposited inside the slit formed by the guide rails, but instead were located at the entrance of the slit (Fig. 1h). Pollen tubes were observed to have germinated from the pollinia (Fig. 1i, j), indicating successful pollen deposition.

Nectar secretion was confirmed in all flowers, with an average volume of 1.98 ± 0.31 µL (*n* = 9).

### Flower visitors and evaluation of pollinators

**Fig. 2:**
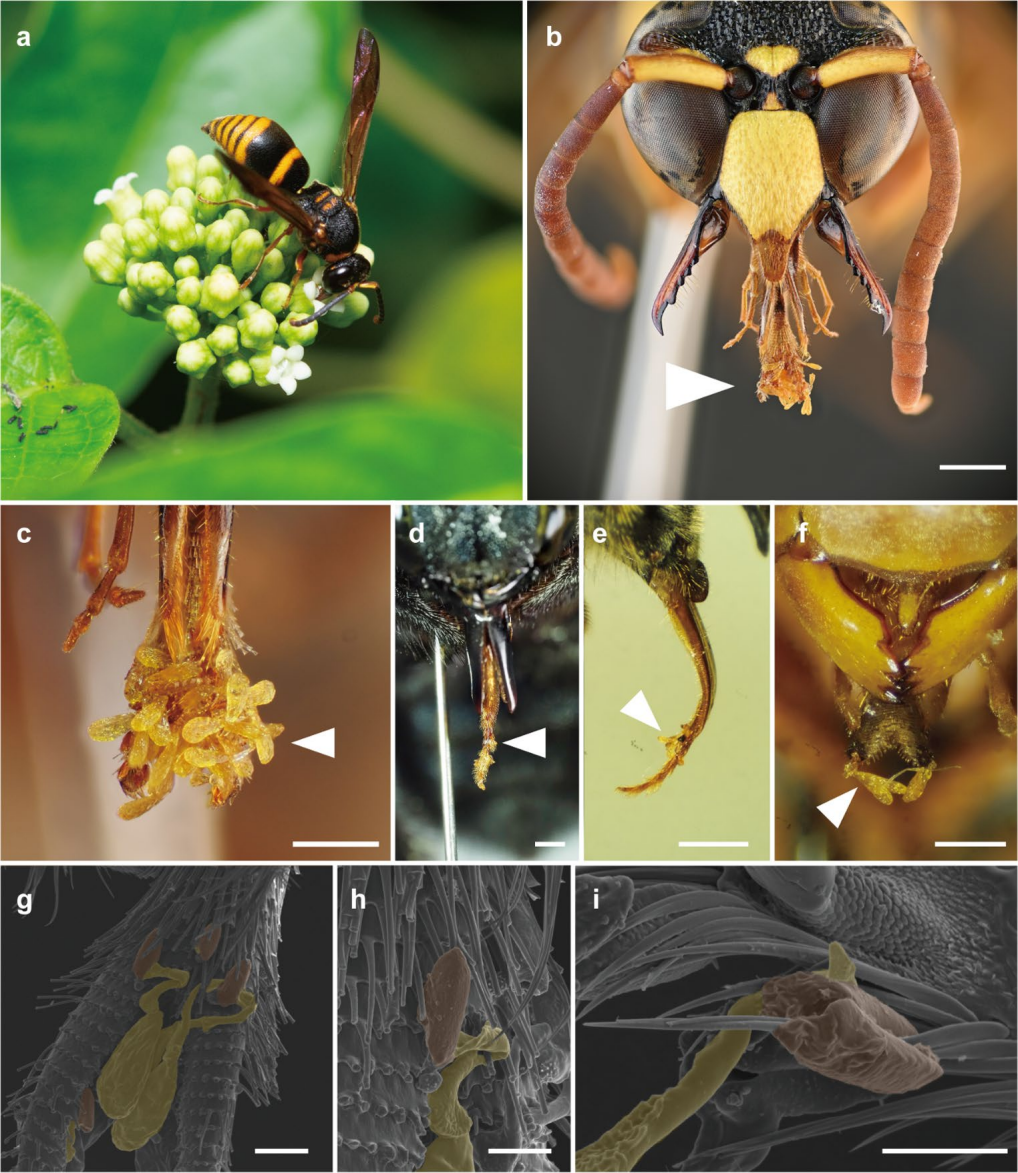
Pollinators and pollinium attachment (triangles). (**a**) *Rhynchium quinquecinctum* feeding on nectar by inserting its mouthparts into the flowers. (**b**) Pollinia attached to the glossa of *Anterhynchium flavomarginatum*. (**c**) Close-up view of glossa. (**d**) *Xylocopa flavifrons*. (**e**) *Apis mellifera*. (**f**) *Polistes jokahamae*. (**g**) Scanning electron microscopy (SEM) image of the glossa of *A*. *flavomarginatum*. (**h**) and (**i**) Corpusculum (clip) attached to glossal hairs in *A*. *flavomarginatum and Apis mellifera*. Scale bars: 1 mm for (**b**), 0.5 mm for (**c**) and (**f**), 1 mm for (**d**) and (**e**), 0.1 mm for (**g**), and 50 μm for (**h**) and (**i**)

**Fig. 3:**
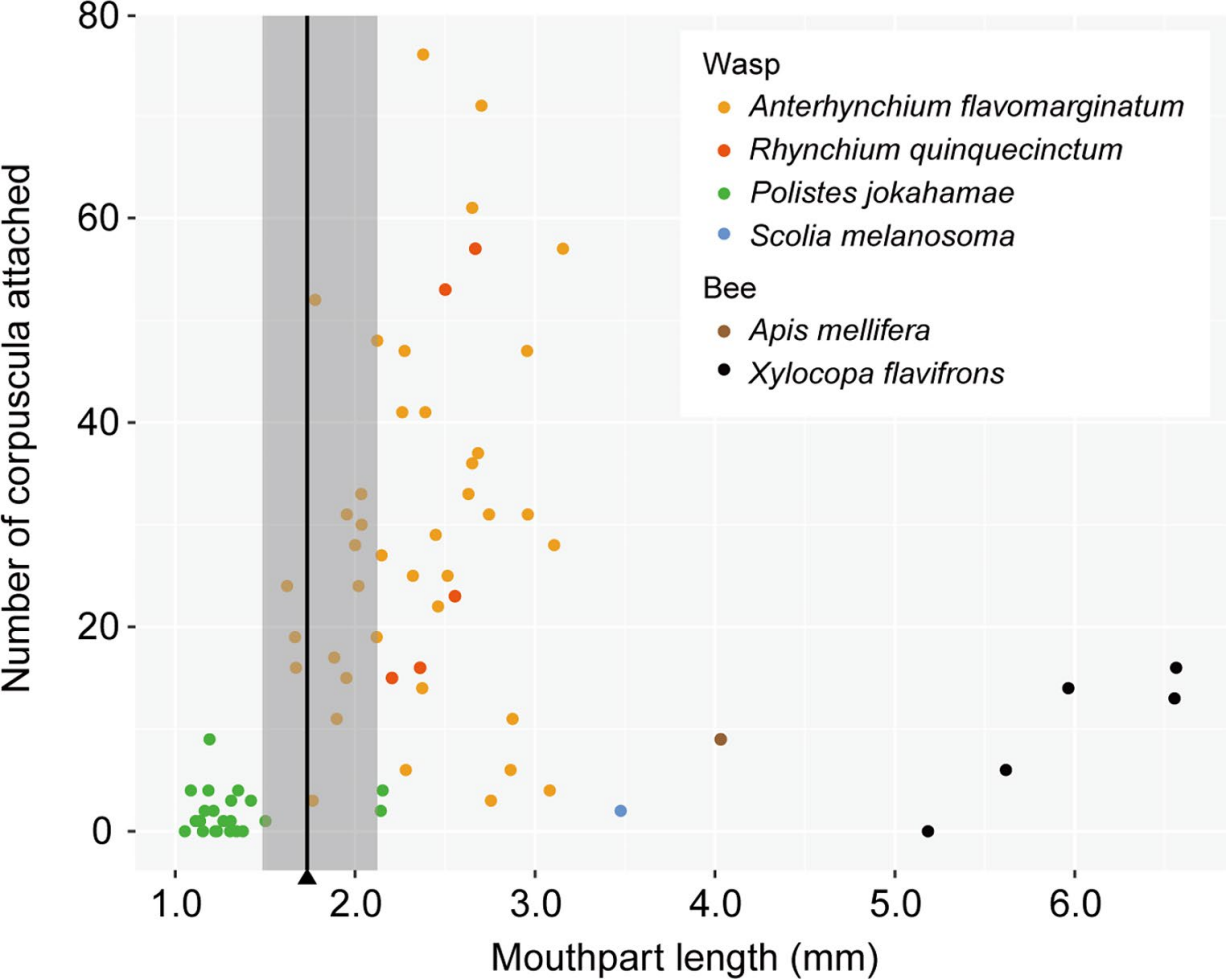
Relationship between the functional length of the mouthpart and the number of corpuscula attached to the glossa. Shaded area indicates the range (minimum to maximum) of flower tube depths; vertical line within this area indicates the average

During 32 h of observation, we recorded 125 floral visitors, 92 of which were captured (Table 1). More than 70% of the visitors (83 individuals) were wasps, with the potter wasp *Anterhynchium flavomarginatum* (Vespidae, Eumeninae) and paper wasp *Polistes jokahamae* (Vespidae, Polistinae) being the predominant species (Table 1). Other visitors included an infrequent potter wasp (*Rhynchium quinquecinctum*) (Fig. 2a), the introduced honeybee *Apis mellifera*, the endemic Okinawan carpenter bee *Xylocopa flavifrons*, the brachodid moth *Nigilgia limata*, flies, and ants, all at lower frequencies than wasps. Pollinia were found attached via the corpuscula to the glossa of six hymenopteran species, including both wasps and bees (Fig. 2b–i). Dipterans had some hairs on the mouthpart, but did not carry pollinia. Lepidopterans did not possess hairs on their mouthparts and did not carry pollen. There was a large difference in the numbers of attached pollinia between taxonomic groups (Table 1; Fig. 3). In the potter wasps *A*. *flavomarginatum* (*n* = 40) and *R*. *quinquecinctum* (*n* = 5), captured individuals carried 29.5 ± 17.9 (mean ± standard deviation [SD]) and 32.8 ± 20.53 corpuscula (Table 1), respectively; the maximum number of corpuscula per individual was 76 (Fig. 3), giving the impression of a swollen glossa (Fig. 2c). The paper wasp *P*. *jokahamae* carried far fewer corpuscula (average, 1.83; *n* = 15; Table 1). Carpenter bees and one honeybee carried 9.8 (*n* = 5) and 9 corpuscula, respectively.

The relationship between mouthpart length and the number of attached pollinia is shown in Fig. 3. The number of pollinia differed among species (ANOVA, F = 12.32, *P* < 0.001). The AIC values of linear models including pollinator species, mouthpart size, and their interaction were 621.6, 659.1, and 621.4, respectively, with the lowest AIC obtained by the interaction model. This model revealed that size did not have a positive effect on the number of attached pollinia (*P* = 0.174). When we focused the analysis on the most frequent visitor, *A*. *flavomarginatum*, there was no correlation between the number of attached pollinia and mouthpart length (*P* = 0.284). The floral tube depth was 1.75 ± 0.16 mm (*n* = 20).

### Pollinator behavior in relation to flower color

**Fig. 4:**
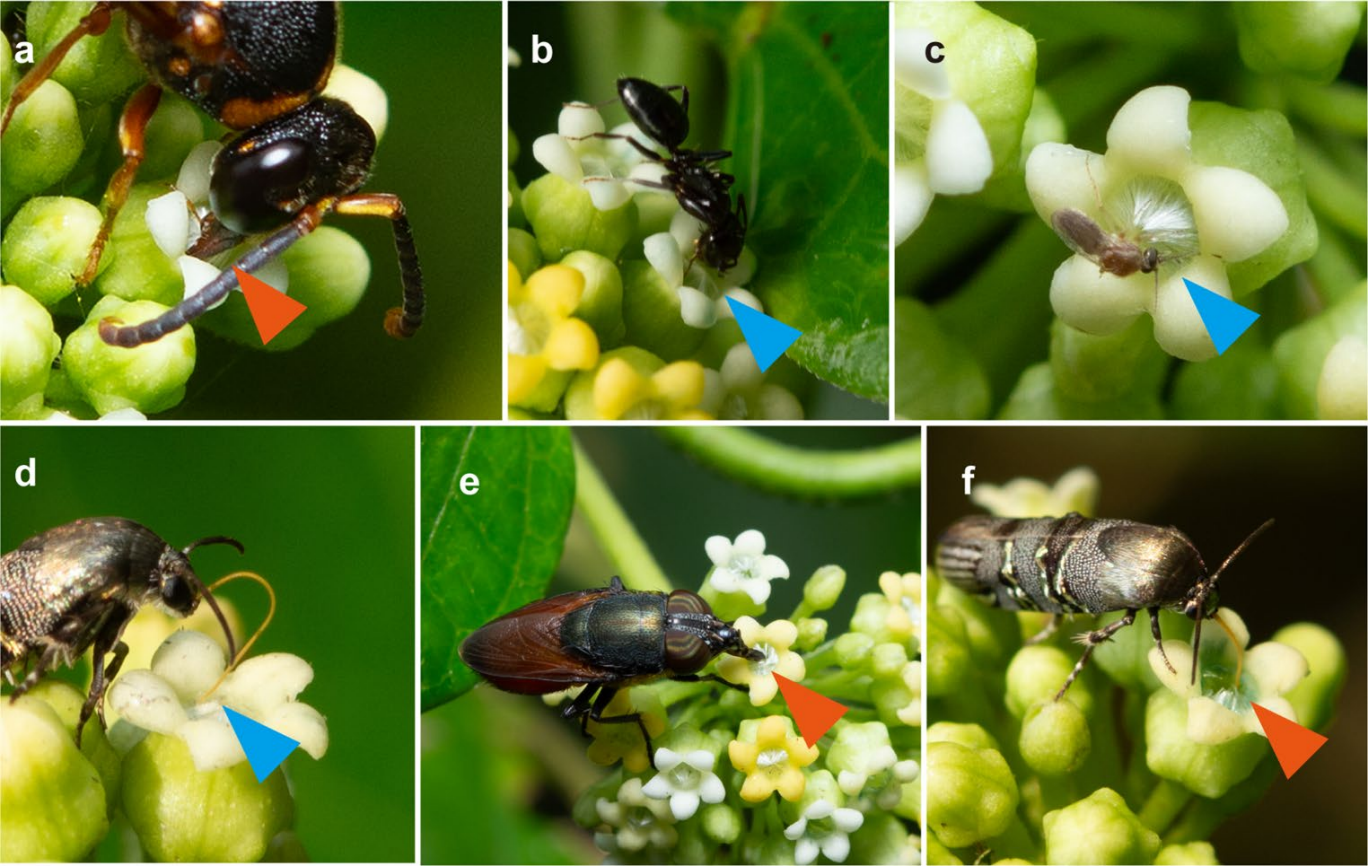
Behavior of floral visitors in relation to floral hairs. Orange and blue triangles indicate the success or failure of mouthpart insertion by an insect. (**a**) Mouthparts of *R*. *quinquecinctum* penetrating floral hairs in a white flower. (**b**) *Camponotus bishamon* and (**c**) Ceratopogonidae sp. attempting to enter flowers. (**d**) The brachodid moth *Nigilgia limata* attempting to insert its proboscis into a white flower. (**e**) *Stomorhina obsolete* and (**f**) *Nigilgia limata* successfully inserting their probosces into yellowish flowers with naturally and manually disturbed hairs, respectively

All floral visitors appeared to be seeking nectar. However, their behavior varied depending on the condition of the hairs at the pollen tube entrance and their taxonomic group. Wasps, carpenter bees, and honeybees exhibited similar behavior, consistently inserting their proboscis through the hairs to access the floral tube (Fig. 4a). By contrast, smaller moths, flies, and ants were often blocked by the hairs and were unable to insert their proboscis, leading them to abandon their attempts (Fig. 4b–d).

Visitation attempts to white flowers were recorded 13 times for moths, 2 times for biting midges, and 5 times for ants. Among these, only one attempt by the moth *Nigilgia limata* successfully resulted in proboscis insertion into the flower tube. In contrast, eight visitation attempts by *N. limata* to yellow flowers were observed, all of which were successful. Additionally, two blow flies were observed inserting their proboscis into the floral tube of a yellow corolla with disheveled hairs (Fig. 4e). When the hairs were bent with tweezers, the moth successfully inserted its proboscis into the flower tube (Fig. 4f).

### Pollen transfer efficiency and floral color

In nearly all sampled flowers (both white and yellow), at least one pollinium was found to have been removed (Table 2). However, the pollinium removal rates were 2.4 ± 1.0 (mean ± SD) in white flowers and 2.9 ± 1.2 in yellow flowers, showing significantly higher removal in yellow flowers (Mann–Whitney U test, *P* = 0.04; Table 2). Similarly, the proportion of pollinated flowers, i.e., those that received at least one pollinium, was 56.7% for white flowers and 80% for yellow flowers. The pollinium deposition rates were 0.9 ± 1.0 for white flowers and 1.7 ± 1.3 for yellow flowers (Table 2), showing a significant difference between flower colors (Mann–Whitney U test, *P* < 0.01).

**Table 2:** Pollen transfer efficiency (PTE) according to *Marsdenia tinctoria* flower color

Flower color	Flowers pollinated (%)	Flowers with pollen removed (%)	Pollinia removed per flower (mean ± SD)	Pollinia inserted per flower (mean ± SD)	PTE
White (*n* = 30)	56.67	100	2.4 ± 1.0	0.9 ± 1.0	0.18
Yellow (*n* = 30)	80	96.67	2.9 ± 1.2	1.7 ± 1.3	0.29

## Discussion

### Pollinators of *M. tinctoria*

We studied the pollinators of *M*. *tinctoria*, the type species of the genus *Marsdenia*, which is characterized by its conspicuous floral hairs (Liede-Schumann et al. 2022), and the function of these hairs by observing floral visitor behavior and pollen transport. Our results suggest that in the studied population during our period of research, this species was primarily pollinated by potter wasps of the Eumeninae and that the floral hairs function to filter out certain visiting insects. This finding represents a novel case of wasp pollination in the genus *Marsdenia* and provides valuable insights into the function of floral hairs. In addition, we observed that the flowers changed color as they matured.

Although a variety of insect taxa were observed visiting the flowers, only hymenopterans were found to carry pollinia. Lepidopterans and dipterans foraged on yellow flowers, most of which still contained pollinaria; however, corpusculum attachment was not observed in these taxa, likely due to the absence of hairs on their mouthparts in Lepidopterans and short proboscis in Dipterans (less than 1 mm). In hymenopteran pollinators, there was no relationship between mouthpart length and floral tube depth. The hymenopteran pollinators, except for *P. jokahamae*, are equipped with mouthparts (glossa) longer than the floral tube so that they could remove and insert pollinia (Fig. 3). Given the wide variation in the amount of pollinia carried by the most frequent visitor, *A*. *flavomarginatum* (Fig. 3), the lower numbers of pollinia and corpuscula carried by carpenter bees and one honeybee (Table 1) are attributed to the limited number of individuals recorded for these taxa. Thus, these hymenopteran species may exhibit similar effectiveness as pollinators; however, wasps were the primary pollinators, being much more abundant than bees (Table 1).

In the studied *M*. *tinctoria* population, nearly 100% of both white and yellow flowers showed evidence of pollinarium removal, and 67% of yellow flowers, which appeared to be at a later blooming stage, had received pollinaria (Table 2), which is a remarkably high rate. According to the PTE calculations, 40% of all pollinia produced were used in pollination, an extraordinarily high rate considering that most members of the Asclepiadoideae have a PTE of around 3–20%, even sometimes zero (Heiduk et al. 2017; Shuttleworth et al. 2017; Mochizuki et al. 2017; but 80% was recorded in *Miraglossum verticillare*: Ollerton et al. 2003). This finding suggests that wasps and other Hymenoptera are extremely effective pollinators in *M*. *tinctoria*. Frequently, a single flower received more than three pollinia, or two pollinaria were found to have been inserted into the same slit (Fig. 1h–j). Pollinia deposited outside the slit germinated pollen tubes that penetrated the slit (Fig. 1i) and multiple pollinia deposited at the entrance of a slit were observed to have germinated pollen tubes. This is the first reported example in Asclepadoideae outside of the genus *Hoya* in which flowers have adapted to achieve successful pollination via pollinia deposition on the guide rails (Mochizuki et al. 2017). It is noted that pollen might compete over stigma in *M. tinctoria*. The number of pollen grains normally contained in a single pollinium exceeds the number of ovules within a single ovary by approximately 1 to 4 times (Wyatt et al. 2000), thus two pollinia are sufficient to fertilize all the ovules of one flower. Therefore, the situation in *M. tinctoria*, where a single stigmatic chamber occasionally receives more than two pollinaria (Fig. 1j), may lead to competition among pollen for fertilization. Although examples of male–male competition in plants are rare and remain poorly understood, pollen competition via the development of competitive pollinarium traits has been recorded among the Asclepiadoideae (Cocucci et al. 2014). Investigating the potential for male competition with a detailed examination of pollen and ovule numbers per pollinium and ovary in *M*. *tinctoria* could provide further insights into its reproductive ecology.

### Function of floral hairs

Floral hairs are commonly observed across plant lineages and are typically associated with functions such as pollinator attraction, reward signaling, or behavior modification (Coombs et al. 2011; da Silva 2022; Tagawa 2023). In this study, hairs deep within the floral tube were concluded to regulate pollinator behavior to ensure efficient pollen transport, as observed in other members of the Asclepiadoideae (Kunze 1991). Although trichomes on leaves are well documented as physiological defenses (Sato and Kudo 2015), the defensive role of floral hairs has not been extensively studied.

The hairs appeared to form a barrier at the entrance of the floral tube, seemingly preventing access to the flower’s interior. Our observations showed that the hairs were neatly aligned in the early stages of blooming, whereas in later-stage, yellow flowers, many of the hairs appeared disordered. In trap flowers, such as those of genus *Aristolochia*, the direction of the hairs changes over time (Oelschlägel et al. 2009); however, this does not appear to occur in *M*. *tinctoria*. Even in yellow flowers, some hairs remained upright (Fig. 1), and in some instances, damage to the hairs was observed after pollinator visits. The floral hairs of *Sisyranthus* appear to serve multiple functions, which vary among species (Ollerton et al. 2003). Due to the limited observations of flower visitors’ behavior in relation to the state of the flower hairs, we must remain aware of the possibility that the hairs of *M. tinctoria* may have functions beyond serving as a barrier against ineffective pollinators.

Smaller visitors such as ants and moths, which did not carry pollinia, were often observed attempting to access nectar but giving up due to the obstruction posed by the floral hairs (Fig. 4b–d). This suggests that the hairs help select which insects can access nectar. In the Asclepiadoideae, pollinarium attachment requires a precise morphological fit (Mochizuki et al. 2017), such that for *M*. *tinctoria*, the floral hairs and structure of the gynostegium act as dual filters for potential pollinators. Some small visitors successfully inserted their probosces into yellow flowers with disordered hairs, which could impose a negative impact via nectar consumption (Fig. 4e, f). Therefore, we conclude that the hairs function primarily to conserve nectar rewards for legitimate pollinators, rather than to select efficient pollen transporters.

### Floral color change

Floral color change is a phenomenon observed across various angiosperm lineages, in which the color of flowers changes either over time or after being visited by pollinators (Weiss 1995). Even after successful pollination, the flowers remain on the plant for a certain period. Several hypotheses have been proposed to explain the adaptive mechanism for retaining aging flowers, including simultaneously increasing the floral display size for long-range pollinator attraction (Ohashi et al. 2015), guiding pollinators to the rewarding flowers when they approach, and facilitating pollen transport by secondary pollinators (Ito et al. 2021).

Because we did not track individual flowers from bud to senescence in this study, the exact process of floral color change in *M*. *tinctoria* remains uncertain. We examined flowers that were white at the end of the day, but did not simultaneously monitor the state of the floral hairs. However, the fact that flowers blooming in the morning had begun to turn yellow inside the bag by evening suggests that floral color change can occur even without pollinator visits. Our overall observations suggest that the flowers open in the morning, begin to turn yellow by evening, turn fully yellow the next morning, and then drop between that night and the following morning.

Our pollen transfer efficiency data indicated that both white and yellow flowers experienced pollen removal and insertion. Notably, the pollen removal rate was 100% even in white flowers (Table 2). However, the higher rates of both removal and insertion observed in older, yellow flowers suggest that both male (pollen removal) and female (pollen reception) functions were unsaturated in the white stage and continued to increase after the floral color change. Among species that exhibit floral color change, the color-changed older flowers are often nonreproductive and rewardless (Ohashi et al. 2015); however, the yellow-petaled *M*. *tinctoria* flowers observed in this study appeared to contribute significantly to pollination.

Dense floral hairs are synapomorphic in the genus *Marsdenia*, whereas floral color change has been observed only in *M*. *tinctoria* to date (Ohashi et al. 2015). In *M*. *tinctoria*, after the floral hairs were damaged by visiting pollinators, the flowers become susceptible to nectar theft (Fig. 3f). Our observations showed that yellow flowers often had disordered hairs and that non-pollinating insects such as small moths were able to obtain nectar from these flowers. Given these observations, it is likely that the nectar content of yellow flowers was generally lower than that of white flowers, which raises the possibility that white petals may be an honest signal of a fresh flower, in which the nectar reward is preserved (Ito et al. 2021).

### Wasp pollination in the Marsdenieae

Pollinator information for the Apocynaceae, which includes the subfamily Asclepiadoideae, is available for only 13% of its species, although it is a large angiosperm family (> 5,000 species) and among the most well-studied groups in pollination research. The tribe Marsdenieae, to which *M. tinctoria* belongs, includes 27 genera and around 740 species. However, Asclepiadeae (106 genera, ca. 1,820 species) has pollinator data available for 245 species (13.46%) and Ceropegieae (46 genera, ca. 790 species) has data for 156 species (19.746%) (Ollerton et al. 2019), whereas Marsdenieae has data for only 21 species (2.8%). This low percentage is primarily due to the lack of pollination data for genus *Hoya* (Landrein et al. 2021; Mochizuki et al. 2017), which significantly contributes to the tribe’s diversity with 350–450 species, and the limited information available for *Marsdenia*, the second largest genus in the tribe with 71 species (Liede-Schumann et al. 2022). Both genera are primarily distributed in Southeast Asia, where research has generally lagged behind other regions worldwide. There are few publications on pollination in the genus *Marsdenia*, but flies, beetles, and moths have been reported as pollinators (Sakagami et al. 2019). This study on *M*. *tinctoria* represents a significant step forward in understanding the pollination systems and floral evolution of tribe Marsdenieae.

Pollination by wasps is relatively rare, although it has independently arisen in various angiosperms such as the Orchidaceae, Scrophulariaceae, and Araliaceae (Willmer 2011). In the Asclepiadoideae, it appears to have independently evolved in the tribe Asclepiadeae, as reported in several genera such as *Miraglossum*, *Pachycarpus*, and *Oxypetalum* (Ollerton et al. 2003; Shuttleworth and Johnson 2009; Vieira and Shepherd 1999). This study provides the first report of wasp pollination in the tribe Marsdenieae, offering an opportunity to explore why wasp pollination has evolved multiple times within Asclepiadoideae.

## Electronic supplementary material

Below is the link to the electronic supplementary material.

Supplementary Material 1

## Data Availability

The data used for statistical analysis of the relationship between mouthpart and floral tube lengths and that of PTE are available via 10.6084/m9.figshare.28235432.v1.
