# Supplementary material for: Wasp pollination and pollinator filtering by dense hairs at the floral tube entrance in Marsdenia tinctoria (Apocynaceae)
Source: J Plant Res. 2025 Feb 20;138(3):447–57. doi: 10.1007/s10265-025-01621-z (PMC12062159; doi:10.1007/s10265-025-01621-z)
Supplement: Supplementary file 1 — Supplementary Material 1 [file 10265_2025_1621_MOESM1_ESM.pdf]

Electronic supplementary materials

Title:

Wasp pollination and pollinator filtering by dense hairs at the floral tube entrance in *Marsdenia tinctoria* (Apocynaceae)

Authors:

Ko Mochizuki, Ayako Watanabe-Taneda

Journal:

Journal of Plant Research

Corresponding author: Ko Mochizuki

(Botanical Gardens, Graduate School of Science, The University of Tokyo, Tokyo 112-0001, Japan)

E-mail: apis3330@gmail.com

Content:

Tables S1: Summary of observations.

Table S1. Summary of observations.

Date	Duration
July 28, 2014	1100-1200
July 26, 2016	1615-1730
July 27, 2016	1030-1930
July 28, 2016	1000-1430
July 29, 2016	1000-2030
August 16, 2018	1700-1800
August 18, 2018	1500-1730
August 19, 2018	0930-1700
